# Low vision and rehabilitation for older people: integrating services into the health care system

**Published:** 2008-06

**Authors:** Liz Simon

**Affiliations:** Christian Blind Mission Low Vision Advisor, PO Box 525, Hluhluwe 3960, South Africa.

**Figure:**
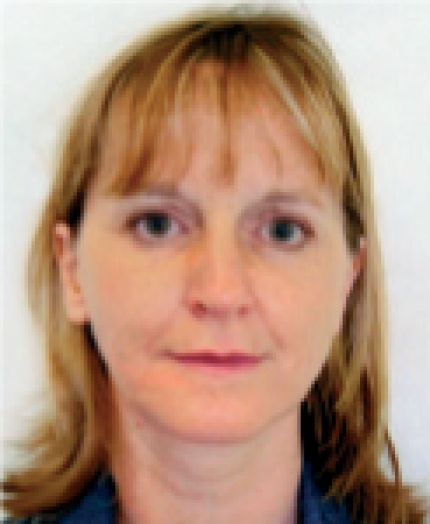


## CASE STUDY : MARY K

Mary K, a 71-year-old widow, had been gradually losing her sight over the past five years. Looking after her five grandchildren had become increasingly difficult. Recently, her neighbour told her about the eye hospital 80 km away; the neighbour had heard of older people who came back from the hospital able to see again.

After much convincing, Mary made plans to go to the eye hospital. She sold three bags of maize and gave her radio to the taxi driver so that he would take her to the hospital. The grandchildren cried when they saw her leave, but her neighbour agreed to look after them for a few days. All this was worth it: after all, she would come back able to see again.

Mary was sick in the taxi and the road took its toll on her old, aching body, but she did not mind because the eye doctor would make her see again. After two hours of waiting in the queue at the eye clinic, Mary was seen by the eye doctor. She was very excited. Dr N examined her eyes and, after what seemed like forever, he told her that she should go back to the village. He was sorry, but there was nothing he could do for her eyes. Mary protested and told him what her neighbour had said, but he just repeated the same words: “I am sorry, there is nothing I can do for you.” Mary walked out of the eye clinic, wondering what to do. No one could help her and no one seemed to care.

Unfortunately, in many parts of the world, the above scenario is all too common in eye clinics. Faced with patients like Mary, eye care practitioners often feel uncomfortable and do not know what to say. Too often, therefore, these patients are turned away when in fact much can be done to help them.

Patients like Mary, who are not completely blind, but whose sight cannot be improved either by treatment (such as cataract surgery) or by the provision of ordinary near or distance spectacles, are said to have 'low vision'.

## What is low vision?

Low vision refers to a permanent loss of vision which makes it difficult for a person to perform many daily activities. A person with low vision presents all three of the following characteristics:

- impairment of visual functioning even after treatment and/or standard refractive correction
- visual acuity ranging from light perception to <6/18 (0.3 logMAR), or a visual field smaller than ten degrees from the point of fixation
- the person uses, or is potentially able to use, vision for the planning and/or execution of a task.^1^

People with low vision are, in principle, capable of using their vision and wish to do so. If given the appropriate low vision devices and training, they do not typically need to use white canes or to learn Braille (although these may benefit some persons with low vision as an additional or complementary support). Many of these patients are not technically considered blind, although they might be classified as such in their country.

For patients with low vision, there is still hope for a better life. This is possible through the use of low vision devices and rehabilitation. **Low vision devices** (such as magnifiers) help patients make the best use of whatever vision is available to them. **Rehabilitation** teaches patients how to adapt their environment appropriately in order to make the best use of their existing vision. Patients who are prescribed low vision devices are also taught how to use these devices in their daily life.

## Low vision in older people

The causes of low vision in older people vary according to region. In general, low vision can be due to glaucoma, diabetes, macular degeneration (which is not present in some parts of Africa), hypertensive retinopathy, or retinal detachment. Cataract cannot be considered a cause of low vision, unless there is a good reason not to perform a cataract operation (by definition, low vision is a permanent loss of vision that cannot benefit from surgical or medical intervention).

Older people with low vision face specific challenges. They have more difficulty getting access to eye care and treatment in general as they are often dependent on their relatives (financially and physically) and tend to be more socially isolated (see articles on pages 24, 26, and 31).

In older people, low vision is usually accompanied by other physical disabilities, as these become more common with increasing age. Disabilities such as hearing or cognitive impairment mean that older people will find it more difficult to understand instructions in a health care setting; they may not hear them properly or may need them to be repeated more often than a younger patient. The boxes on pages 27 and 32 provide some guidance on how to cater for the needs of older people in a health care setting.

Physical disabilities may also influence the suitability of certain low vision devices for older patients. For example, whereas children with low vision may find it easy to hold reading materials very close to their eyes, this might be much more uncomfortable or tiring for older people.

It is important to cater for the specific needs of older people when setting up or managing low vision services - not least because they represent the vast majority of low vision patients who will be seen by eye care workers. Indeed, it is estimated that 80 per cent (48 million) of all people who need low vision care are aged over 50.^1^

## Low vision services - an integrated approach

Low vision services are delivered by a very broad range of disciplines and social systems throughout the world, but all with one purpose in mind: improving patients' use of their available and functional vision.

Rather than create new systems to provide low vision care and rehabilitation to older persons, it is much better to focus instead on developing services that can be integrated into existing eye care systems. This is known as an **integrated service delivery approach** to low vision care.

One glove does not fit all, since different countries have different medical, social, and rehabilitation systems. However, it should be possible to integrate low vision services for older people into each level of care (Table 1, overleaf), and into the rehabilitation services of the country, where these are available.

**Table 1:** Provision of low vision services at different levels of care as part of an integrated service delivery approach

Level of care	Who is involved	Services provided
Community level	Community-based rehabilitation workers, community case finders, community leaders, and consumer advocates	• Raising awareness • Identification and referral/transfer • Basic home-based rehabilitation
Primary level (e.g. clinics in the community)	Primary health care workers and primary eye care workers	• Raising awareness • Identification and referral/transfer • Basic rehabilitation (discussion of options only)
Secondary level (e.g. a district hospital)	Ophthalmic nurses, optometric technicians, and ophthalmic clinical officers	Low vision assessment: • Diagnosis • Prescription of optical and non-optical low vision devices (low and medium power) • Instruction in the use of low vision devices • Counselling • Basic rehabilitation (discussion of options only)
Tertiary level (e.g. a provincial or regional referral hospital or teaching hospital)	Ophthalmic nurses, optometric technicians, ophthalmic clinical officers, optometrists, ophthalmologists, low vision therapists, orientation and mobility instructors, occupational therapists, social workers, and researchers	Same as the secondary level, plus: • Prescription of high-power and complex low vision devices • Training and support for eye care practitioners at other levels • Rehabilitation training • Research

It is estimated that 30 per cent of older people with low vision can be assisted at community and primary level, and that 50 per cent can be assisted at secondary level. The remaining 20 per cent will need tertiary level services.^2^

Most patients will first be seen at the secondary level and be referred from there. It is important to have very clear, well-defined referral mechanisms between the different levels of the health system, no matter where patients first report. Referral routes should not be rigid or inflexible, as referral should depend on the needs of individuals.

**Figure:**
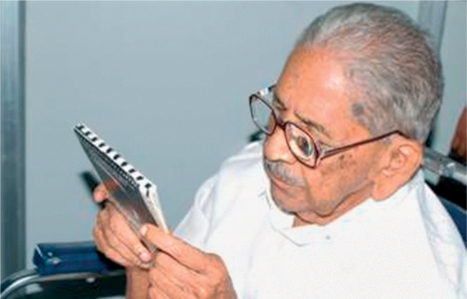
**An 81-year-old former magistrate, diagnosed with age-related macular degeneration, is able to read again after receiving 24-dioptre low vision spectacles. INDIA**

The following paragraphs provide tips on how to integrate low vision services at different levels of the health mated care system.

### Community level

Community-based rehabilitation (CBR) workers, community case finders, community leaders, and consumer advocates can do the following at the community level:

- **Raise awareness** in the community so that people know what low vision is, that there is hope, and that something can be done even for those who cannot benefit from surgery or the provision of spectacles.
- **Identify and refer/transfer:** identify older people in the community who can beneft from low vision services, refer them to these services, and make sure they get to these services (i.e. are successfully transferred). Older persons may need physical assistance when travelling.
- **Provide basic home-based rehabilitation:** spend time in the homes of older people with low vision; help family members or carers to make life easier and safer for the person with low vision. For example, safety can be improved by encouraging family members to keep the house tidy, to keep knives in a safe place, and to make the fire in a separate area. Life can also be made easier for the person with low vision by working with colour and contrast. For example, serve rice in a dark bowl or place a border of brightly painted stones along the path leading to the home.

### Primary level

At primary level, primary health care workers and primary eye care workers will also raise awareness; they can identify older people with low vision and refer or transfer them.

Since they will see only patients who come into the clinic, the rehabilitation they can offer will not be home-based. Instead, it will involve talking to the person with low vision and their family about what they can do.

### Secondary level

Ophthalmic nurses, optometric technicians, and ophthalmic clinical officers will usually be the first to provide a thorough assessment of a person suspected of having low vision (see box below).

### Tertiary level

Eye care practitioners at tertiary level (see list in Table 1) can prescribe high-power and complex low vision devices, but otherwise they provide much the same services as at secondary level, with the following additions:

- **Orientation and mobility:** training in Braille and the use of a cane for people who cannot be helped with low vision devices (or who have very low vision); training family members and carers in the ‘sighted guide’ technique (how to walk with a person who is blind or has low vision).
- **Training and support for eye care practitioners at other levels:** specialists are usually based at tertiary level, where they act as instructors and train other eye care practitioners.
- **Rehabilitation training:** some eye care practitioners at tertiary level have special expertise in environmental modifications (which involve working with colour and contrast in the environment of the person with low vision) and can teach ‘activities of daily living’ (ADL) skills, such as how people with low vision can wash their clothes.
- **Research:** any research in low vision and rehabilitation will usually be based primarily at a tertiary institution.

**Figure:**
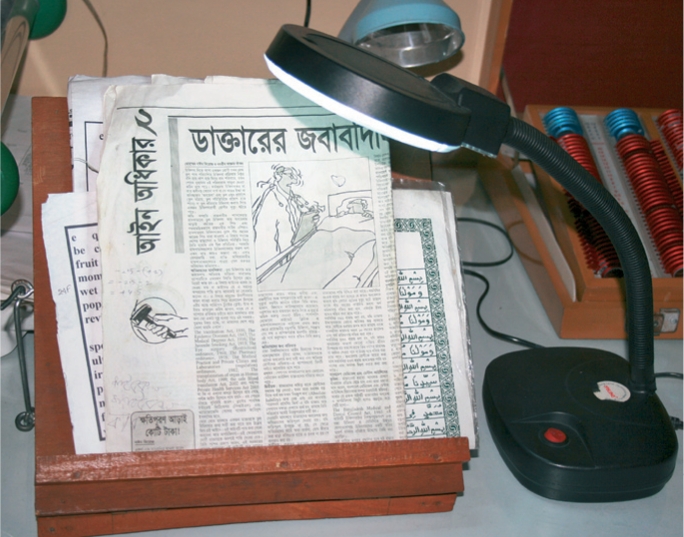
**A reading stand and angled lamp. Both are non-optical low vision devices.**

## Providing appropriate services

The importance of providing appropriate low vision and rehabilitation services cannot be overemphasised. Every person with low vision - especially older people - must be considered on an individual basis.

The needs of older patients with low vision will depend on their circumstances: the region they come from, their economic status, their literacy levels, their family responsibilities, their attitude towards ageing and disability, their general health, their motivation, and so on. Whereas loss of reading ability is often considered to be the most devastating consequence of visual impairment in high-income countries, it may have little significance and impact on the quality of life of an older person in a rural village in a low- or middle-income country.

Low vision clinicians may be tempted to prescribe an array of magnifiers, telescopes, filters, or non-optical aids. However, if these devices are unsuitable, they may simply end up under the mattress when the patient returns home. The clinician must therefore ensure that the low vision devices they prescribe are acceptable in the home; the older patient must also be motivated or interested enough to use them.

## The importance of training to meet low vision needs

As populations live longer, many countries must prepare for great increases in the number of people with age-related conditions resulting in low vision. In the future, integrated low vision and rehabilitation services for older people will assume more importance.

There is currently a lack of skills relevant to the care and rehabilitation of older people with low vision. We have seen how different people in the health system can provide different components of low vision services.

We need to act now and train the relevant practitioners (eye care and other); develop and include low vision services in existing eye care systems; and create awareness amongst all medical, social, and rehabilitation services to ensure that patients like Mary are not sent home with no promise of help.

**Low vision assessment**

1. When conducting a low vision assessment for an older person, be patient and understanding, and take your time. Make sure the assessment takes place in an environment that facilitates communication (see article on page 31).
2. It is important to listen very carefully to what is said: your task is to try and find out what the person really wants out of the assessment (how they want to be able to use their vision) and to do your best to help them achieve that.
3. A good assessment should include:
  - distance and near visual acuity testing
  - contrast sensitivity testing
  - good refraction
  - assessment of magnification needs
  - prescription of low- and medium-power low vision devices, both optical and non-optical (such as an angled desk, an angled light, or writing guides), for distance and near vision
  - instruction in the use of low vision devices
  - counselling to explain the extent of, and reasons for, the person's low vision and to help the person accept their situation and make the best of it
  - a discussion of rehabilitation - what the person and their family can do to make life easier and safer.

**MARY K: A BETTER OUTCOME**

Perhaps all that Mary actually needed to go home happy was to be told why her vision could not be restored and that a community-based rehabilitation worker would make a home visit. This person would have demonstrated to Mary what adaptations could be made to her home, allowing her to make the best use of her vision. Mary could also have been helped if someone had informed her of any social welfare grants she would be entitled to.

If Mary needed to see things near to her, she may have benefited from getting a simple magnifier to check the grandchildren's feet for chiggers, take stones out of rice, or read (if she were literate).

However, being reassured that she would not go completely blind, and that help was available, is perhaps the greatest service Mary could have received.
